# Caring alone: older adult care burdens, practices, and structural imbalances among China’s one-child generation

**DOI:** 10.3389/fpubh.2025.1694636

**Published:** 2025-12-15

**Authors:** Shuai Xiang, Hongxu Xiang, Qiao Ren, Qinwen Deng

**Affiliations:** 1 School of Public and Management, Guangxi University, Nanning, China; 2 School of Health and Wellness, Chongqing Polytechnic Institute, Chongqing, China; 3 School of Law, Guangxi University, Nanning, China; 4 School of Law, Chongqing University, Chongqing, China

**Keywords:** China, one-child per couple policy, only children, aging, older adult care burdens, qualitative research, grounded theory

## Abstract

**Background:**

Between 1980 and 2015, China’s one-child policy reshaped family structures. Now, as this generation enters adulthood and their parents age, the only children face the daunting challenge of supporting multiple older parents in an increasingly aging society.

**Purpose:**

This study focuses on the practical challenges and needs faced by China’s one-child generation in caring for their aging parents. By examining their caregiving experiences, the study aims to reveal the multiple pressures they bear and to provide policy- and system-level insights for alleviating the older adult care burden on this unique generation.

**Method:**

This study is a qualitative analysis based on content from social media. A grounded theory analysis of online discussions was conducted using NVivo 14.0 (QSR International). Focusing on questions such as “What happens as the parents of one-child families grow old, and who will ensure their care?,” our study systematically gathered and coded discussions from Zhihu, a major social media platform in China.

**Results:**

The Chinese only children in this study’s sample face intense older adult care pressure, including economic strain, labor shortages, and emotional exhaustion. They struggle to balance caregiving with career development while shouldering dual pressures of supporting both aging parents and young children. Structural inequalities—such as the gap between government promises and actual social security, and disparities between urban and rural areas, as well as within and outside the formal system—worsen the challenge. At the same time, the weakening of traditional filial piety deepens cultural and psychological strains, further fragmenting intergenerational caregiving responsibilities.

**Discussion:**

Research indicates that the issue of older adult care for parents of only children in the sample requires not only the reconstruction of a fairer and more sustainable older adult care ethic across society but also urgent institutional efforts to promote more inclusive and equitable support for older adult care and medical services. Meanwhile, the sample observations indicate that the development of technology and the establishment of a high-quality, affordable older adult care service system will become important pathways to easing the caregiving pressure of the one-child generation and providing greater social support.

*“I don’t dare to be poor, I don’t dare to get sick, I don’t dare to marry far away, and I don’t dare to die—because my parents have only me.” — Zhihu user*

## 1 Introduction

Population aging is a global phenomenon. At present, China has become one of the countries with the largest older population and the fastest pace of aging in the world (1, 2). According to data released by the National Bureau of Statistics in 2024, China has already entered a moderately aged society, and the trend of population aging is irreversible (3, 4).

At the end of 1979, the Chinese government introduced the one-child policy, which became an important factor contributing to the intensification of population aging (5). The one-child policy refers to the regulation that each married couple in China was permitted to have only one-child (6). In the context of the one-child policy, local authorities implemented aggressive fertility control measures (7). Women who became pregnant beyond the permitted quota were subjected to forced abortions, while those who had already given birth faced compulsory sterilization (5, 8). Families that exceeded the birth limit were also subjected to punitive measures that threatened their livelihoods, such as fertility penalties (social support fees several times the household income), loss of public employment, and other sanctions (9, 10). The one-child policy has led to a prolonged decline in China’s fertility rate and a continuous increase in the proportion of the older population (11). In October 2015, driven by concerns over population aging, the Chinese government announced the end of the one-child policy^1^. However, the one-child policy, which lasted for 36 years, not only had a profound impact on social consciousness and family structures but also posed significant challenges to patterns of caregiving. A large number of so-called ‘4-2-1’ (12) or ‘4-2-2’ (13) families emerged, in which a couple must not only raise one or two children of their own, but also support four aging parents. Compared with the situations in other countries where having only one-child is an individual family choice, the one-child phenomenon in China was a state-driven structural social experiment in population control. The long-term and stringent one-child policy, which covered hundreds of millions of people in China, has created the largest population of only children in the world (14). Due to its policy-driven nature and massive demographic scale, China’s one-child generation has become an important lens through which to examine the tension between individual responsibility and institutional structures. It also provides a meaningful case for understanding family transformation, intergenerational relationships, and the ethics of care.

How the parents in one-child families will be supported has long been a central concern for gerontology researchers in China (15–17). The caregiving role of China’s only children has drawn attention in fields such as nursing and sociology (18–21). According to Articles 13–14 of the “Law of the People’s Republic of China on the Protection of the Rights and Interests of older persons (2018),” older adult care is to be family-based, and adult children bear a legal obligation to care for their aging parents. Therefore, in a context of legal mandates, limited social support, and a strong cultural emphasis on filial piety, China’s only children typically bear primary responsibility for caring for their parents (20). As early as 2000, scholars had predicted that in the coming decades, the lives of older parents of only children would become a pressing social issue (22). A scholar has pointed out that between 2023 and 2032, China’s only children will begin to confront, on a large scale, the practical challenges of caring for their aging parents (23). A simulated quantitative study indicates that, compared with non-only children, China’s only-child caregivers bear greater caregiving challenges and stress (19). This entails physical strain and financial difficulties, as well as irreplaceable legal obligations and psychological challenges (20). At present, clarifying the experiences of China’s only children as caregivers holds profound academic value and enduring practical significance (23).

This study analyzes online discussions by China’s one-child generation about caring for their parents, aiming to explore the challenges they face and their reflections. The first generation of only children in China was born around 1980 and is now in their forties (5). As both the workforce of society and bearers of family responsibilities, they are simultaneously entering the life stage of caring for older parents and raising their own children (24). In this context, they face increasingly complex—and at times conflicting—roles (25, 26). As only children’s parents begin to experience functional decline and enter a stage of heightened care needs, the nature of caregiving shifts from emotional companionship and financial support to more demanding physical care and long-term medical management (23, 27). This shift gives rise to a new stage of circumstances, in which the limited caregiving capacity of only children competes with the increasingly demanding care needs of their parents. When the one-child policy was implemented, the official government slogan promised: “One-child is best; the government will take care of the parents’ old age (28).” A systematic understanding remains lacking regarding how this generation perceives the government’s fulfillment of its promises. The everyday lives of these individuals as caregivers, along with the challenges they encounter and the coping strategies they adopt, remain underexplored in the literature. More importantly, we need to understand how they, as firsthand participants, reflect on China’s government policies, social support systems, and the future trajectory of older adult care. These questions form the core of this study, which aims to illuminate the activities and critical reflections of the one-child generation as they navigate the dual pressures of family and society.

Social media is a virtual community platform where people increasingly share and exchange opinions, ideas, and experiences. With the development of the internet, social media has become an important channel for obtaining information and influencing reality, as well as a key research site in fields such as sociology and gerontology (29, 30). Grounded theory is particularly suitable for qualitative analysis of the vast and easily accessible discussions found online. Therefore, this study applies grounded theory to examine online content related to “the only children caregiving for older parents” in China (31). The study is guided by three primary objectives: Firstly, to construct a three-level coding framework to present the real-life context, action logic, and reflections of China’s only children in providing older adult care. Secondly, to use this framework to deepen the theoretical understanding of caregiving by China’s only children within the fields of gerontology, nursing, and public health. Finally, by revealing the real-life challenges reflected in the study, to provide practical insights for improving social support services and the social security system.

## 2 Materials and methods

### 2.1 Research methods

In 1967, Glaser and Strauss jointly developed grounded theory and applied it to research in the social sciences (32). Since then, the grounded theory approach has been widely applied across multiple disciplines, including anthropology, psychology, gerontology, nursing, and business (29, 33, 34). As a representative method of qualitative research, grounded theory emphasizes identifying, developing, and integrating theory from data collected in the field (35). This bottom-up approach to theory development aims to minimize the influence of prior knowledge and researchers’ subjective biases (36). It is a theory generated from the data, rather than a theory formulated prior to the data (36).

From the perspective of grounded theory’s conceptual framework, it is particularly suitable for research in relatively underexplored areas (34). Social phenomena that have recently emerged and sparked widespread discussion and attention fall into this category. Due to their brief existence, the connotations and scope of such newly emerged social phenomena remain unclear. At the same time, the abundance of social discussion facilitates the collection of firsthand data on specific aspects of socio-cultural life, making grounded theory particularly well-suited for exploratory research.

Currently, research on the care of older parents by China’s one-child generation is limited, there is a lack of a more comprehensive conceptual framework and understanding. Given that China’s one-child generation has already begun to face the tangible pressures of caring for their aging parents (24, 37), discussions about the challenges they encounter have increased year by year. At the same time, many individuals share their personal experiences on public online platforms. Therefore, as a newly emerging phenomenon rich in firsthand data, this study is particularly well-suited for the application of grounded theory.

### 2.2 Data sources and collection

#### 2.2.1 Data sources

This study aims to conduct an in-depth exploration of the lived experiences of the contemporary Chinese one-child generation in the process of caring for their aging parents. This study utilized qualitative data collected from the online Q&A community — Zhihu. Zhihu is China’s leading community platform for text-based Q&A discussions, and many qualitative studies have drawn data from it (29, 33). Zhihu encourages users to elaborate on their personal experiences and opinions in detail through long-form posts, within a serious, professional, and friendly atmosphere. Unlike short-video platforms such as Douyin (the Chinese version of TikTok) and Kuaishou, Zhihu provides a large amount of high-quality, original text content that is easily accessible. Zhihu has nearly 100 million monthly active users (38). Based on user demographics, Zhihu’s users are primarily young and middle-aged adults aged 24–40, which aligns with the target population of this study (39). They have engaged in extensive discussions on topics related to “the one-child generation providing care for their parents” on the Zhihu platform, making it an ideal data source for this study.

#### 2.2.2 Inclusion and exclusion criteria

The research team systematically searched for questions and answers containing keywords such as “only-children,” “parents,” and “older adult care” to ensure both accuracy and richness of the information collected. We focused on the top three questions on Zhihu in terms of number of discussions and popularity, which were: “How can the only children take good care of both parents in the future?”; “What should the one-child generation do when their parents grow old?,” and “How can the only children provide older adult care for both parents?” These three sets of questions and their related discussions span a period of 13 years, from July 2012 to July 2025. During this period, a total of 1,090 posts were generated, containing rich information with a combined word count of 450,000 words.

After applying strict selection criteria, a total of 495 posts, comprising approximately 191,000 words, were included in the analysis. The selection criteria were as follows:

1 Identity criterion: Individuals from one-child families who are about to assume or are currently assuming responsibility for their parents’ older adult care. Based on the content of the responses, eligible participants were categorized into the following three types.

Type 1: The only children who provide older adult care for their parents serve as the primary source of information for this study.
Type 2: The spouses of only children, whether they are only children themselves or not, share responsibility for caring for the parents of a one-child family. Although their responses represent a smaller proportion, they enrich the study by providing perspectives from both the family members and spouses.
Type 3: Only children who provide older adult care for their grandparents. For various reasons, they assume some of their parents’ responsibilities and care for their grandparents. In the process, they accumulate substantial insights and experiences related to ‘caring for parents.’ As the lifespan of older adults continues to increase, the size of this group is gradually expanding, and their responses are worthy of inclusion in the study.

Using the identity criterion, we excluded responses from groups such as older adult care industry professionals, parents from one-child families, and non-one-child families, thereby refining the focus on the study’s target population.

2 Content quality criteria: Priority was given to entries that provided detailed personal experiences, emotional responses, and reflections on parental older adult care. Responses that were vague or unrelated to the research topic were excluded.

The screened sample provided rich data for subsequent qualitative coding and analysis. The collected data were processed and analyzed using NVivo 14 Plus. The research team coded the data, compared and merged similar responses, and identified emotions, themes, and conceptual frameworks.

### 2.3 Data analysis processes

**Figure 1:**
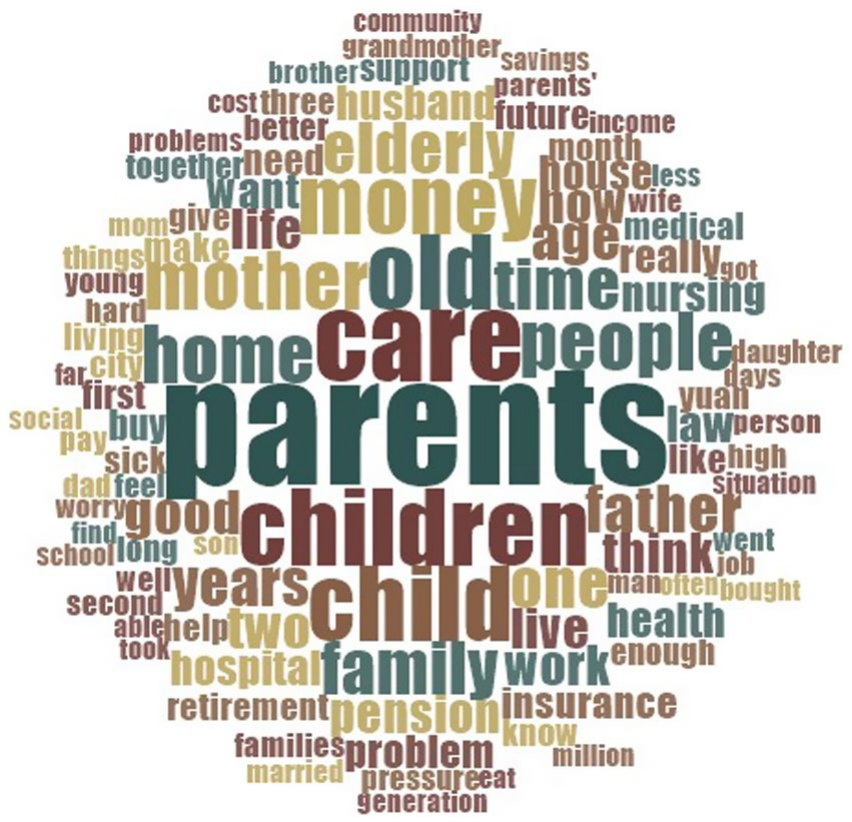
Grounded word cloud.

This study employs a qualitative research approach, relying on the research strategies of grounded theory to examine issues related to contemporary Chinese only children providing older adult care for their parents. Before beginning the coding process, we first preprocessed the textual data using NVivo 14 Plus. Using the word frequency analysis function, we generated a high-frequency word cloud (see Figure 1). Figure 1 illustrates the main content of the textual data in this study.

Coding is central to grounded theory analysis and serves as a key means for understanding the thoughts, perspectives, and responses of the information providers regarding the research topic (40). In this study, we followed the coding procedures of grounded theory and conducted material analysis in three stages: open coding, axial coding, and selective coding. The core of the coding process lies in ensuring the rigor and consistency of the coding procedures, to meet the standards of validity and reliability in qualitative research (41). To enhance consistency in judgment, we adopted a model combining individual coding in Excel with group discussions for consolidation. First, each researcher in the team conducted open coding of the original text in their individual Excel files, using phrases and labels to describe meanings. Then, the results were consolidated. Through team meetings, the researchers discussed and compared each file, and on this basis reached a group consensus to establish a unified coding scheme.

#### 2.3.1 Open coding

Open coding is the first stage of grounded theory analysis, during which the research team identifies and categorizes different concepts and themes. In this process, the team carefully reads the posts line by line, marking key words, concepts, and emotions, while identifying potential units of meaning. At this stage, we developed both thematic codes and emotional codes.

In the thematic coding stage, we engaged in repeated discussions and readings. We identified 45 thematic codes related to the current situation of only children caring for their parents, their views and expectations about the future, and issues concerning China’s social security and older adult care system, such as “psychological burden,” “physical burden,” and “financial strain.”

For ease of comparison and to distinguish emotional coding from thematic coding, we applied a five-point Likert scale to code emotions. The five categories were “Strongly negative,” “Mildly negative,” “Neutral,” “Mildly positive,” and “Strongly positive,” with the emotional expressions in the text displayed along this continuum from low to high.

#### 2.3.2 Axial coding

Axial coding represents the second level of coding. Building on the emergent themes identified during open coding, axial coding further refines, aligns, and categorizes these themes to form distinct thematic categories and prepare for selective coding (36).

At this stage, the research team organized and connected the concepts obtained from open coding, identifying the logical relationships among them, such as causality, identity, context, and coping strategies. Through continuous review and reflection, the codes were screened and integrated, allowing the themes to become progressively stable, clear, and distinct from one another. In this process, the research team stayed close to the data, explored the connections between different themes, examined and clarified their relationships, and thereby constructed a persuasive and comprehensive categorical system.

Through continuous comparison and analysis, we integrated the nodes from open coding and reclassified them into 13 basic categories.

#### 2.3.3 Selective coding

Selective coding is the final step, focusing on the core categories to develop a comprehensive explanatory model. It represents the research team’s effort to move toward the specific focus of the study, aiming to develop and create an explanatory framework and ultimately construct meaning. Through this process, we identified three core categories.

### 2.4 Data credibility and interpretive validity

#### 2.4.1 Researchers triangulation

To enhance the data credibility and the interpretative validity, this study adopted researcher triangulation during the data analysis process (42). Four researchers independently coded the sample content, followed by group discussions in which discrepancies were repeatedly compared and examined until a consensus was reached.

#### 2.4.2 Contextual judgment

When reading the posts, the researchers assessed their authenticity and the richness of information by considering the poster’s self-reported life experiences, caregiving context, comment interactions, and previous posts.

#### 2.4.3 Coding prevalence comparison

To ensure the prevalence of the extracted concepts, the research team compared data from Zhihu with relevant discussions on other Chinese social media platforms. The main themes and concepts were found to be consistent across platforms, further supporting the credibility of the data interpretation.

### 2.5 Coding saturation test and ethical statement

#### 2.5.1 Coding saturation test

Theoretical saturation is a point at which no new properties can be derived from subsequent data (34). During coding, we retained 25% of the original textual data to assess theoretical saturation. In the coding test of the final 25% of the data, no new attributes, dimensions, or relationships emerged, indicating that theoretical saturation was achieved. Therefore, we conclude that all nodes in this study are sufficiently dense, and the relationships among nodes are adequately described, reaching theoretical saturation (36).

#### 2.5.2 Ethical statement

The data for this study were obtained from open forums, and their use within the parameters of public data is ethically appropriate. Furthermore, posts and replies on Zhihu are freely accessible to anyone. The data used in this study were strictly in compliance with the Zhihu User Agreement. The part of this study involving human participants was reviewed and approved by the Medical Ethics Committee of Guangxi University (Approval No. GXU-2025-090).

### 2.6 Potential bias and reflexivity

In terms of potential bias, all four authors were born during the period of China’s one-child policy. Two of the authors are only children, while the other two are not. Three of the authors have experience assisting their parents in caring for functionally impaired grandparents, while one author has up to 2 years of regular volunteer experience in community older adult care and wellness services. This background provides the research team with heightened sensitivity to the challenges faced by caregivers. To minimize potential biases arising from personal experiences in interpreting the data, the research team engaged in collective discussions, reflective practices, and cross-checking throughout the analysis process, striving to ensure the diversity and objectivity of the analysis.

## 3 Results

**Figure 2:**
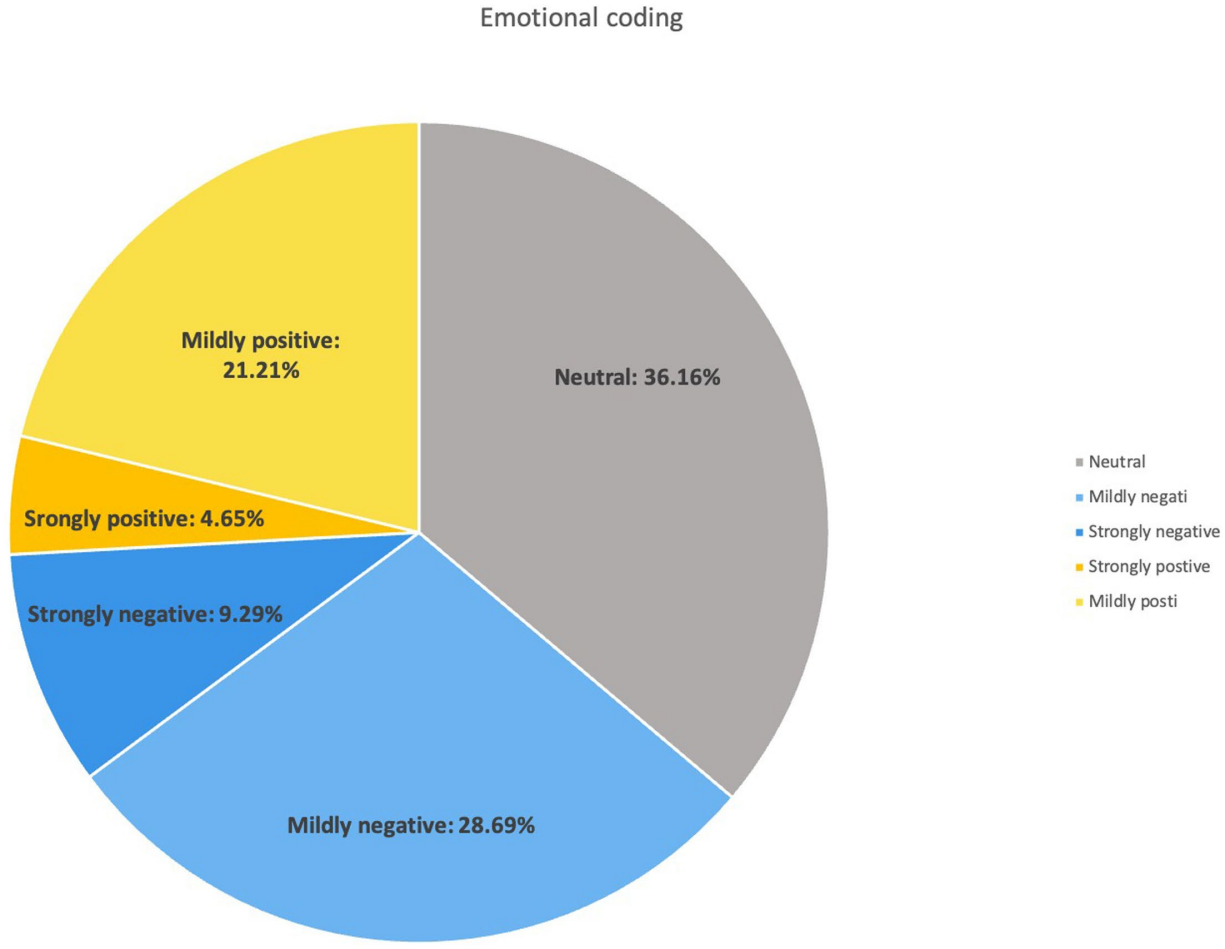
Emotional coding.

This study presents the emotional tendencies of Chinese only children, primarily urban and well-educated, who are active online, regarding the care of their aging parents (Figure 2), showing an overall trend that is neutral to somewhat negative. We categorized the emotional tendencies of the 495 responses included in the study on a five-point Likert scale, ranging from low to high. Responses coded as “Strongly negative” reflected the lowest emotional state, with participants explicitly expressing intense negative emotions such as “extreme anxiety, suicidal thoughts, and severe distress” (46 participants, 9.29%). Those coded as “Mildly negative” were in the next lowest emotional range, expressing negative feelings such as “anxiety, depression, fear, and helplessness” (142 participants, 28.69%). Participants coded as “Neutral” exhibited calmness in their responses, showing no particular emotional tendency (179 participants, 36.16%). Those coded as “Mildly positive” believed that with the coordination of factors such as “technology, earning money, and insurance arrangements,” older adult care issues could be relatively well managed (105 participants, 21.21%). Finally, participants coded as “Strongly positive” represented the most relaxed group in the study, perceiving no burden in caring for their parents and even considering that their parents could provide them with solid support (23 participants, 4.65%). The data above indicate that the largest proportion of participants held a negative attitude (37.96%), followed by those with a neutral attitude (36.16%), while the smallest proportion were optimistic (25.86%).

In the thematic coding section, selected open codes (Table 1) and all three levels of coding (Table 2) are presented in tabular form. To enhance the transparency of the research process, Table 2 presents the number of codes for each category at each level. Overall, the results of the core coding in this study yielded three main thematic categories: “challenges,” “actions,” and “reflections.” Due to Zhihu’s privacy protection policies, this study was unable to obtain the characteristics of the posters. In the following, we present the open-coded excerpts in italics with double quotation marks.

**Table 1:** Open coding (excerpt).

Original texts (excerpt)	Free node (excerpt)
I am currently an undergraduate, so it is not yet my responsibility to provide older adult care. However, whenever I think about this issue, I feel afraid.	a1 Feels anxious and fearful about the future.
Because my grandmother recently fell ill, and it is still uncertain whether it is cancer, I feel very distressed. At the same time, I am preparing for the graduate school entrance exam and attending classes, with no one to confide in. Suddenly, I realize that the helplessness of being an only child often comes to the fore when family members encounter difficulties.	a2 Current stress leading to pessimism and a sense of helplessness.
I am not yet financially independent, and having my parents bear such a heavy burden makes me feel guilty.	a3 Guilt and self-blame as a child.
I am already caught in this predicament. I just graduated from university, have not started working or gotten married, and my father suffered a cerebral thrombosis, which left him paralyzed, incontinent, unable to speak, and dependent on care. I have been taking care of him at home, while my mother has been absent for several years. Being an only child, there is no one to help me. I cannot work outside because my entry-level salary would not cover the cost of my father’s nursing home. I do not know what to do, so I have been taking care of my father at home for a year and a half. Beyond financial difficulties, the greatest harm to me has been physical and mental: the long-term strain of caregiving, the isolation from being unable to leave home, all of which have severely affected my mental health.	a4 Psychological fatigue resulting from long-term caregiving burdens.
During that period, I felt every day that I might get into a car accident; I could even fall asleep while waiting at a red light.	a5 Chronic fatigue and sleep deprivation.
By the time my grandfather was discharged from the hospital, only child was suffering from a lumbar pain flare-up and bronchitis, while the other had a stomach problem and could not get out of bed—this is not an exaggeration.	a6 Acute illnesses resulting from caregiving for the older parents.
In our small family, the most noticeable issue is not the lack of money, but the lack of people.	a7 Insufficient family manpower support.
However, I cannot help but think: “They only have me as their daughter.” If I move to another city for work and settle there, who will take care of them when they become frail or ill in a few years? On the other hand, if I stay by their side, it seems I cannot fulfill my desire to explore the world, nor can I balance work and caregiving effectively.	a8 Conflict between personal development (work/studies) and caregiving responsibilities.
Parents and children. How to choose between one’s own parents and one’s children—perhaps this is not the “right” way to phrase it, but in reality, there are many situations where the choices feel inherently wrong.	a9 Conflict between caring for parents and caring for one’s own children.

**Table 2:** The three-level coding system.

Open coding	Axial coding	Selective coding
Initial category	Main category	Core category
A1 Fear and anxiety about the future (39)	B1 Psychological burden (94)	C1 Practical challenges of older adult care responsibilities (284)
A2 Helplessness and sadness resulting from current pressures (22)		
A3 Guilt toward parents (17)		
A4 Psychological fatigue caused by long-term caregiving burdens (16)		
A5 Chronic fatigue (3)	B2 Physical burden (8)	
A6 Onset of acute illnesses (5)		
A7 Insufficient family caregiving manpower (40)	B3 Shortage of caregiving manpower (137)	
A8 Difficulty balancing personal development and caregiving (40)		
A9 Intergenerational caregiving conflicts (27)		
A10 Parental dependence on children and resistance to external caregivers (17)		
A11 Scarcity of high-quality care resources (13)		
A12 Medical and medication expenses (24)	B4 Financial strain (45)	
A13 Costs of caregiving services (14)		
A14 Reduced household income (7)		
A15 Sufficient income and cash as the core (92)	B5 Active resource preparation (227)	C2 Individual actions for older adult care (463)
A16 Comprehensive pension and insurance arrangements (59)		
A17 Co-residence to integrate resources (76)		
A18 Maintain physical health and actively manage personal risks (19)	B6 Physical and mental preparedness (44)	
A19 Adjust mindset to accept challenges (25)		
A20 Preference for next generation not to be only children (14)	B7 The replanning of the children’s generation (26)	
A21 Educate the next generation for rational older adult care (4)		
A22 Engage their children to assist in caring for grandparents (8)		
A23 Expectation management to reduce psychological gaps (9)	B8 Guidance for older parents (63)	
A24 Long-term health management (25)		
A25 Cultivate elders’ adaptability to modern technology (4)		
A26 Guide elders to accept professional older adult care services (11)		
A27 Encourage elders to enrich their own lives (14)		
A28 Marry a non-only-child to share caregiving burden (8)	B9 Personal life planning (103)	
A29 Work and marry locally (22)		
A30 Remain unmarried and childless (17)		
A31 Provide full-time care for parents (15)		
A32 Mutual assistance with relatives (18)		
A33 Hire caregivers or housekeepers for assistance (23)		
A34 Care crisis and intergenerational inequity caused by policies (29)	B10 Views on the one-child policy (88)	C3 Reflections on policies and culture, and visions for the future of older adult care (291)
A35 Fewer family conflicts and better economic conditions for only children (45)		
A36 Demanding that the government fulfill its commitments (14)		
A37 Aging before wealth, coupled with the lack of a comprehensive older adult care system (11)	B11 Evaluation of aging-related policies and welfare systems (95)	
A38 Primary healthcare and older adult care facilities in grassroots communities need improvement (13)		
A39 Benefits and compensation of civil servants and public institution employees (48)		
A40 Benefits and compensation of ordinary urban workers and rural residents (23)		
A41 Children’s sacrifices under the influence of filial piety (17)	B12 Critique of Confucian older adult care culture (35)	
A42 Incompatibility with modern family concepts (11)		
A43 Rejection of diversified older adult care approaches (7)		
A44 Openness to euthanasia (9)	B13 Vision for the future of older adult care (73)	
A44 Technology-driven smart older adult care (15)		
A45 Silver economy promoting more comprehensive older adult care services (49)		

The number shown in parentheses () reflects the number of codes for each grounded node.

### 3.1 Practical challenges of older adult care responsibilities

This study’s participants face multiple real-life challenges, including psychological pressure and physical burden, compounded by a shortage of caregiving manpower and financial strain.

#### 3.1.1 Psychological burden

The coding results indicate that the sources of psychological burden for the participants in this study are complex, mainly consisting of the following four aspects. These include a fear of future caregiving responsibilities and a sense of helplessness when confronted with current challenges. On the one hand, they often feel guilty for being unable to adequately care for their parents due to limited capacity; on the other hand, they may experience prolonged caregiving fatigue, which can ultimately lead to psychological breakdown.

##### 3.1.1.1 Fear and anxiety about the future

The posters expressed feelings of fear about the future, and many people feel that they are not yet prepared. Concerns about their parents’ illness were the primary triggers of their anxiety:

*“What I fear most is precisely this, because it feels unsolvable—I can only pray that my parents remain safe and healthy, without any major illnesses.”*

##### 3.1.1.2 Helplessness and sadness resulting from current pressures

Some posters shared the pressures they are currently facing. Their unique family structures mean they lack siblings to share the responsibilities, and when caregiving burdens fall solely on themselves, feelings of pessimism and helplessness arise:

*“Having said all this, the pressure on only children is indeed overwhelming. Between caring for my parents and managing academic stress, I feel as if I’m being forced into premature aging—it’s truly exhausting.”*

##### 3.1.1.3 Guilt toward parents

Many of the posters in the discussion expressed guilt about their parents. To avoid placing the caregiving burden on their only daughter, one poster said her parents planned to move to a nursing home, aiming to “give the best to their child without leaving any trouble behind.” The poster, worried about being unable to care for her parents adequately, felt deep guilt in the face of parents’ selfless devotion:

*“At this moment, tears welled up. My parents devoted their entire lives to raising me, yet I am mediocre, have little to make them proud, and even neglected my graduate exam preparation due to a poor mindset. I feel utterly incapable. Yet they are still thinking about not causing me any trouble in the future. The reality feels suffocating. I truly blame myself.”*

##### 3.1.1.4 Psychological fatigue caused by long-term caregiving burdens

One poster shared their experience of falling into caregiver burnout after long-term care for their ill mother. Within just 1 year, the poster’s mother was successively diagnosed with uterine fibroids and meningioma, and underwent a craniotomy. The poster falls into typically psychological fatigue.

*“In a large hospital 200 kilometers away from home, I slept every night on an air mattress in a corner of the ward. Although caring for my mother did not require much physical effort, I was deeply distressed. As a young person spending every day with patients, I felt I could never be happy again.”*

#### 3.1.2 Physical burden

Compared with the 94 coded items for psychological burden, the physical burden reflected in the Zhihu discussions was much lighter, with only 8 coded items. However, it is worth noting that instances of physical burden have already begun to emerge. These primarily manifest as chronic fatigue and acute illnesses caused by caregiving responsibilities.

##### 3.1.2.1 Chronic fatigue

A poster shared his experience. Because caregiving requires intense focus and sustained effort, he experienced prolonged physical and mental exhaustion when his parents fell ill consecutively:

*“In 2016, my parents fell ill one after another and were hospitalized. I had to take leave to care for them, and almost all major decisions, such as signing for surgeries, rested on me alone. The physical and psychological torment was nearly unbearable.”*

##### 3.1.2.2 Onset of acute illnesses

The spouse of a one-child poster shared her husband’s experience of suffering an acute illness due to the overload of caregiving:

*“My father-in-law, suffering from dementia, was unable to care for himself, while my mother-in-law, who was partly self-sufficient but extremely difficult to deal with, added further strain. Although my husband was generally in good health, the combined stress of work and caregiving led to the onset of gastric illness.”*

#### 3.1.3 Shortage of caregiving manpower

In one-child families, the shortage of caregiving labor is a particularly acute problem.

##### 3.1.3.1 Insufficient family caregiving manpower

Due to the inherent limitations of the “4-2-1” family structure, many respondents indicated they generally lack sufficient familial caregiving support. Many responses pointed out that a shortage of caregivers is more urgent than a shortage of money. As one respondent noted:

*“Although finances are not an issue, chronic illnesses requiring constant care are the most exhausting. Even when professional caregivers are available, many tasks still require family members to be personally involved. My father used to suffer from epilepsy, and my grandmother broke her bones three times—each time it was a physically and emotionally draining ordeal.”*

##### 3.1.3.2 Difficulty balancing personal development and caregiving

Many respondents mentioned that caring for their parents inevitably consumes personal time and energy, and this conflict becomes especially pronounced when children live far from their hometowns. In their replies, they expressed concerns about having to interrupt their studies or resign from work to return home and care for their parents:

*“If my mother were to become paralyzed, would I have to give up the career I have painstakingly built in Beijing, abandon the work I love, and return to my hometown to care for her? And if, after my mother’s paralysis, my father were also to fall ill, what should I do then? I have imagined countless unfavorable outcomes, each one tightly bound to my destiny and future.”*

##### 3.1.3.3 Intergenerational caregiving conflicts

Some respondents mentioned that, upon reaching middle age, pressures from both older adult care and childcare gradually arise. They felt it was difficult to balance caregiving responsibilities between their parents and their children:

*“Watching my son grow day by day, I became acutely aware that I cannot have it all: to be both a good husband and father, and a devoted filial child.”*

##### 3.1.3.4 Parental dependence on children and resistance to external caregivers

Some responses indicated that their parents were overly dependent on their children for care and resisted the involvement of “non-family caregivers” such as nannies or professional caregivers:

*“From my father’s surgery a year and a half ago to the current recurrence and ongoing treatment, I have been by his side almost continuously day and night. My father is psychologically very dependent on me; even when his physical condition allows, he expresses the desire for my presence. Therefore, I remain in the hospital around the clock until physically unable to continue.”*

##### 3.1.3.5 Scarcity of high-quality care resources

The scarcity of high-quality caregivers was also cited in Zhihu discussions as a major reason for the heavy caregiving burden. Some respondents expressed concerns that in China, caregivers tend to have low levels of professional training and that caregiving costs are relatively high:

*“Hiring a caregiver is unavoidable, but high-quality caregivers are scarce and expensive. Moreover, I believe it will become increasingly difficult for the post-1980s and post-1990s generations to hire caregivers, as fewer people are willing to do this work and the population is declining, driving labor costs higher.”*

#### 3.1.4 Financial strain

In the data, respondents commonly reported that serious and major illnesses have a devastating impact on household finances, simultaneously leading to substantial increases in expenditures and reductions in income.

##### 3.1.4.1 Medical and medication expenses

Respondents believed that for families with only one-child, minor illnesses or injuries rarely cause financial strain, but serious conditions such as cancer, paralysis, or Alzheimer can impose a heavy burden:

*“If it weren’t for major illnesses, caring for my parents would not be a problem. After all, health insurance covers expenses, and our retirement funds are sufficient for living. But major illnesses have shown me the true impact of medical costs.”*

##### 3.1.4.2 Costs of caregiving services

Some respondents pointed out that caregiving expenses have become a heavy burden, particularly when the rehabilitation and therapy period is long and professional nursing care is required:

*“The growing financial burden is immense, as two people have to cover the older adult care costs for four individuals. Whether hiring caregivers or placing them in nursing homes, the expenses are considerable and beyond what an average family can bear.”*

##### 3.1.4.3 Reduced household income

From the discussions among Zhihu users, it is evident that illness-induced poverty still exists in China. Respondents noted that when parents require long-term care, their working hours and opportunities are reduced, and some even quit their jobs to provide full-time care, leading to a decline in the overall household income:

*“I truly feel that the future older adult care burden on me is overwhelming! Government support for pensions and medical expenses is minimal for non-employees, and older adult care will consume most of my future income!”*

### 3.2 Individual actions for older adult care

Based on the aforementioned difficulties, the participants in this study actively made plans and arrangements to cope with the challenges of their parents’ aging. Their action focus on five main areas: resource accumulation, physical and psychological preparedness, replanning intergenerational responsibilities for the next generation, proactive guidance and education for their parents, and personal life planning.

#### 3.2.1 Active resource preparation

Resource preparation primarily consists of three components: cash savings, pension or retirement insurance, and housing.

##### 3.2.1.1 Sufficient income and cash as the core

Most respondents believed that working hard and earning money at a young age is key to addressing these challenges. As one participant noted:

*“As long as the finances are in place, many pressures can be alleviated—everyone would agree on this. Spending a little less each year and saving some money seems reasonable, as life is long and it is necessary to set aside funds to lay the foundation for the future.”*

##### 3.2.1.2 Comprehensive pension and insurance arrangements

Plenty of respondents emphasized the importance of improving their parents’ security systems, including medical insurance, social security, commercial insurance, and pension schemes. The response of one poster reflected her practical concerns about older adult care and risk prevention:

*“It is essential to implement basic risk management. Given the longer average life expectancy, the likelihood of a family encountering serious illnesses is not negligible, so one should not rely on luck. Purchase social insurance, plan for commercial insurance early, and provide the older population with supplemental accident and cancer coverage.”*

##### 3.2.1.3 Co-residence to integrate resources

A great many respondents mentioned that they planned to bring their parents to live with them (including overseas) in order to consolidate caregiving resources:

*“Living together as much as possible is important. When parents have just retired, they can help take care of the grandchildren, and when they grow older, it is more convenient to care for them. Ideally, buy two apartments, either on separate floors or next to each other.”*

#### 3.2.2 Physical and mental preparedness

Adequate preparation for parental older adult care requires both good physical health and a positive mental attitude.

##### 3.2.2.1 Maintain physical health and actively manage personal risks

Facing the pressures of caring for their aging parents, the only children in the responses recognized the importance of managing their own health and personal risks:

*“After my mother’s recent illness, while I was running around taking care of her, I kept telling myself: take it slow, stay calm, don’t panic. I am the sole pillar of the family, so ensuring my own safety comes first.”*

##### 3.2.2.2 Adjust mindset to accept challenges

Respondents also mentioned that they engage in self-reflection, adjust their mental state, accept issues inherited from the one-child policy, and face current and future challenges with equanimity:

*“Many only children perceive future older adult care as exceptionally daunting, and some cannot even imagine how they will cope—issues are generally manageable while parents are still independent, but the real difficulty arises if parents lose their autonomy. I believe that every problem has a solution; some seemingly unsolvable issues may become clear when viewed from a different perspective.”*

#### 3.2.3 The replanning of the children’s generation

Based on their experiences providing older adult care for their parents, the only children in Zhihu Q&A also reflected on their own future aging, which in turn influences their planning for the next generation.

##### 3.2.3.1 Preference for next generation not to be only children

Part of the respondents indicated that they would have two or more children in order to reduce the future caregiving burden on their offspring:

*“I really want to have two children. I feel that being an only child is too lonely, and I am truly afraid of seeing my child helpless when facing older adult care in the future.”*

##### 3.2.3.2 Educate the next generation for rational older adult care

In this process, many reflected on their own aging and mortality, and hoped to lead by example to guide the next generation in approaching older adult care rationally:

*“I want to set an example for future generations. One should strive to live a meaningful life, but when facing death, be brave and decisive, in accordance with the natural order.”*

##### 3.2.3.3 Engage their children to assist in caring for grandparents

Due to the heavy burden of family caregiving, some respondents stated that they would pass on the caregiving pressure to the next generation and involve their grandchildren in caring for their grandparents:

*“My son is already 14. When my parents reach 70, my son will be an adult and can help me. So, I don’t feel the pressure is overwhelming.”*

#### 3.2.4 Guidance for older parents

In Zhihu discussions, posters believed that guiding their older parents and getting them to cooperate with their actions was very important.

##### 3.2.4.1 Expectation management to reduce psychological gaps

A large number of respondents believed that elders’ expectations should be managed in advance, keeping their desires moderate to reduce the burden on their children. One respondent stated that while elders should maintain a cheerful and contented attitude, they also need to lower their expectations and cooperate with their children:

*“In fact, as an only-child couple, aside from arranging for parents to live nearby, hiring competent caregivers, visiting them as often as possible, and encouraging them to find their own sources of enjoyment rather than relying solely on children and grandchildren for happiness, there is no other solution. Everyone’s emotional journey is their own; ultimately, one must first take responsibility for oneself before being able to care for others. All love should not be based on a sense of sacrifice.”*

##### 3.2.4.2 Long-term health management

Respondents generally indicated that supervising their parents to maintain a healthy lifestyle, cultivate good habits, and undergo regular medical check-ups could reduce the risk of illness:

*“Constantly paying attention to parents’ health, guiding them to abandon unhealthy habits, and encouraging regular exercise. Small check-ups every six months and comprehensive exams annually are essential. As I always say, early detection enables early treatment.”*

##### 3.2.4.3 Cultivate elders’ adaptability to modern technology

Responses mentioned that encouraging older parents to adapt to modern technologies, such as smart home devices and remote older adult care services, helped them reintegrate into society, facilitated communication with their grandchildren, and maintained a youthful mindset,

*“Cultivate the habit of using tools. While parents are still willing to accept new things, continuously introduce and experiment with various technologies—for example, teaching them to use WeChat, FaceTime, and so on.”*

##### 3.2.4.4 Guide elders to accept professional older adult care services

In the early stage of old age, respondents indicated that guiding and transforming their parents’ attitudes toward older adult care—especially regarding professional care services—was very important, as it helped eliminate elders’ prejudices:

“*Enroll parents in day-care facilities early and appropriately. Similar to sending a child to kindergarten, parents may initially be unhappy about staying in a care facility, but we cannot provide 24/7 care ourselves. Two days a month, or a few days in case of emergencies, is manageable. Once familiar, parents become less resistant, much like children attending early education classes.”*

##### 3.2.4.5 Encourage elders to enrich their own lives

The only children who made the posts believed that after retirement, parents should focus on their own lives, develop personal interests, and maintain social networks. As one participant described:

*“She (my mother) enrolled in a senior university, regularly plays table tennis, chess, and darts, attends gatherings with her middle school classmates to sing and socialize, goes shopping and has meals with her close friends, and plays cards with neighbors.”*

#### 3.2.5 Personal life planning

Numerous respondents stated that in order to fulfill their responsibility of caring for their parents, they had adjusted their personal life plans, including aspects such as employment, settlement, marriage, and childbirth.

##### 3.2.5.1 Marry a non-only-child to share caregiving burden

Several only children and their parents in the responses believed that, when choosing a marriage partner, it was preferable to select someone who was not an only child in order to share the caregiving burden:

*“When choosing a partner, it is important that your spouse has siblings, so they are not primarily responsible for older adult care. If they are also an only child, they must have significant advantages in other areas; otherwise, older adult care becomes a serious issue.”*

##### 3.2.5.2 Work and marry locally

To better care for their parents, some respondents stated that they would choose to return to their hometowns to settle, find employment, and marry. The respondents and their parents generally held strongly negative attitudes toward marrying far from home:

*“Only children should not marry far away; it is selfish to spend the rest of your life like that. Work locally, live near home, and when my mom gets old, I’ll just move in with her to take care of her.”*

##### 3.2.5.3 Remain unmarried and childless

A few responses mentioned that the pressure of caring for their parents was so great that they could not afford to raise children. When they felt a lack of energy, time, or money, they chose to reduce or postpone marriage and childbirth in order to fully cope with their parents’ older adult care needs:

*“To resolve this issue, I have decided to remove ‘having children’ from my plans.”*

Certain participants even stated that they chose not to marry in order to avoid the responsibility of caring for their spouse’s parents.

*“Unmarried, I am currently very fearful of marriage, because it would likely mean taking care of two sets of older parents plus children.”*

##### 3.2.5.4 Provide full-time care for parents

Part of the responses mentioned that it has become increasingly common for parents to pay their children to provide full-time care for them. This arrangement was said to occur in families where the parents are financially stable, the children have low willingness to work, and there is a shortage of caregiving labor. As one poster described:

*“It has become common here for the spouse with the lower salary to move all four older parents into one household, managing their care daily as if going to work. The four elders contribute part of their pension to pay their daughter or son, and the son-in-law or daughter-in-law. All six family members will benefit.”*

##### 3.2.5.5 Mutual assistance with relatives

Some respondents mentioned that most of their parents’ generation were not only children, and that relying on cousins for peer support was very important:

*“When we were young, we agreed with our cousins to help each other. Since we are all only children, we have continued this practice over the years, taking turns staying overnight when a family has an issue.”*

##### 3.2.5.6 Hire caregivers or housekeepers for assistance

The only children in the responses held a positive attitude toward hiring caregivers and domestic helpers. They recognized that it was difficult to care for their parents alone and proactively employed caregivers to share the burden:

*“When the family can afford it, we hire a nanny or part-time helper for our parents, depending on their health, to share some daily chores.”*

### 3.3 Reflections on policies and culture, and visions for the future of older adult care

In confronting the burdens of older adult care, only children in Zhihu Q&A also reflected on policies, institutional arrangements, and filial cultural norms, while envisioning the future of older adult care.

#### 3.3.1 Views on the one-child policy

Individuals of different social statuses exhibit markedly divergent views on the one-child policy, which constitutes a primary point of contention in the discussions.

##### 3.3.1.1 Care crisis and intergenerational inequity caused by policies

Certain respondents described only children as “a sacrificed generation,” with both the children and their parents being victims of historical policies.

*“Looking at the world and all of humanity, the one-child policy over the past 30 years in our country is unprecedented in human history. The accumulated problems are severe! The ‘high-risk’ nature of one-child families lies in their most unstable ‘inverted triangle’ structure, which places the entire burden on the only child. In middle age, only children fear hearing about their parents’ illnesses, as a parent’s sickness is like a ticking time bomb, ready to destroy years of a family’s efforts and savings. Without siblings or a fallback, only children must bear two to three times the economic pressure.”*

##### 3.3.1.2 Fewer family conflicts and better economic conditions for only children

A portion of the respondents took a more relaxed attitude toward this. They believed that families where the one-child policy was strictly enforced were mostly public-sector households, and therefore generally had better financial conditions, making it feasible to care for their parents in old age:

*“Only children in these families do not seem to face many difficulties; their economic status is clearly better because regions and populations where family planning was strictly enforced often had secure employment—civil servants, doctors, teachers, and state-owned enterprise employees. These families can afford caregivers and frequently have access to quality nursing homes.”*

Compared with families with multiple children, part of the respondents believed that in only-child families, the responsibility is concentrated, which actually results in fewer disputes over older adult care:

*“I don’t think one-child families face many difficulties. I have seen enough cases in multi-child families where responsibilities are shirked, elders are cared for separately, and disputes arise over inheritance, and so on.”*

##### 3.3.1.3 Demanding that the government fulfill its commitments

Several respondents referred to the slogans used when the government implemented the one-child policy, emphasizing that the government should fulfill its original promises rather than treating them as mere political rhetoric. They believed that being left without support in old age is not the children’s responsibility, but the government’s responsibility:

*“Perhaps the government will introduce corresponding policies for this large group of only children! After all, the slogan at the time was: ‘One-child is best; the government will take care of the parents’ old age’.”*

#### 3.3.2 Evaluation of aging-related policies and welfare systems

Respondents generally believed that China currently faces the challenge of “aging before becoming wealthy” and lacks a comprehensive older adult care system. The quality of healthcare and older adult care institutions serving the older population is relatively low. Regarding pensions and medical insurance, evaluations vary considerably among civil servants, urban employees, and rural residents.

##### 3.3.2.1 Aging before wealth, coupled with the lack of a comprehensive older adult care system

Respondents generally considered China’s social security system inadequate and urban–rural and regional development uneven, leading them to hold a pessimistic view of future older adult care:

*“During the past thirty years of rapid development, our country has relied mainly on technological input and demographic dividends, yet it has not reached mature self-sustaining growth—in plain terms, aging has begun before achieving affluence. What will happen in the future?”*

##### 3.3.2.2 Primary healthcare and older adult care facilities in grassroots communities need improvement

Scores of posts expressed concern and dissatisfaction with the conditions of primary healthcare and social older adult care institutions. The following extreme metaphor provided by a Zhihu user, to some extent, reflects people’s anger and emotions:

*“Considering the current service levels of nursing homes, sending one’s parents there is almost like sending living people to a concentration camp—albeit at a cost.”*

##### 3.3.2.3 Benefits and compensation of civil servants and public institution employees

A majority of responses from only children of families in the civil service and public institutions indicated that, due to their parents’ generous older adult care provisions, they faced almost no financial pressure and were even still receiving financial support from their parents. Their only concern was that they spent too little time with them:

*“Both of my parents are civil servants. After retirement, their combined pension exceeds 20,000 yuan. They own two debt-free properties in a third-tier city, with rental income of 2,500 yuan per month. During their careers, their workplaces fully contributed to medical insurance, annuities, and social security, so medical costs are minimal; even the expenses for imported targeted medications are easily affordable.”*

##### 3.3.2.4 Benefits and compensation of ordinary urban workers and rural residents

Responses from rural families indicated that their parents’ pension levels were low and insufficient to cover basic living expenses, and that all of their parents’ living and medical costs were fully subsidized by the children:

*“I am a 1989-born only child; my parents are farmers—father born in 1961, mother in 1962. Here’s our situation: each parent receives just over 100 yuan per month in pension. After having children, we brought my parents to live with us. My mother stopped working to take care of the child, and my father found a job as a security guard earning 1,800 yuan per month.”*

Responses from urban worker families indicated that their parents’ pensions were higher than those of rural residents, but could only barely support independent living while in good health. Once chronic or serious illnesses occurred, medical expenses had to be covered by the children:

*“There are so many workers, and later the companies failed—this kind of situation is quite common around me. In a fourth-tier small city, parents aged 65–70 have a combined pension of less than 6,000 yuan and only a small self-owned home. If they remain healthy, living independently is possible, but any chronic or major illness depends on the children’s financial capacity.”*

#### 3.3.3 Critique of Confucian older adult care culture

Confucian culture emphasizes that “filial piety ranks first among all virtues,” regarding the care of one’s parents as the child’s moral duty and primary responsibility. Under the modern social context, the drawbacks of this cultural model have gradually become apparent, drawing criticism from many responses in Zhihu discussions.

##### 3.3.3.1 Children’s sacrifices under the influence of filial piety

In the responses, many children began to emphasize individual development and self-realization, opposing excessive personal sacrifice. They engaged in profound reflection and critique of the traditional filial norms that demand children unconditionally fulfill their parents’ wishes:

*“I feel that the issue of older adult care is not merely a matter of money or personnel, but a conflict between our traditional moral values and the modern economic structure—it is fundamentally a question of mindset. According to traditional views, it is taken as self-evident that filial piety for children to stay by their parents’ side and care for them until the end of life, which considers the matter from the parents’ perspective. From the child’s perspective, however, asking a person in their prime to abandon their career, adjust to an older adult lifestyle, and undertake work that is destined to be hopeless—only to grow old themselves after their parents pass away—is itself an extremely harsh expectation.”*

##### 3.3.3.2 Incompatibility with modern family concepts

Some respondents believed that their lives emphasized individualism and that traditional filial norms no longer fit modern families. In Zhihu discussions, many expressed dissatisfactions with the emotional manipulation and moral pressure associated with filial piety:

*“In today’s society, this is the reality. I believe that parents first need to adapt to this situation, learn to live their own lives, and enjoy their days; placing excessive expectations on their children is unwise. We should adopt a different approach, looking at family and parent–child relationships through values similar to those in Western contexts, which is healthier.”*

##### 3.3.3.3 Rejection of diversified older adult care approaches

Certain respondents pointed out that Confucian culture lacks the concept of socialized older adult care, and that nursing homes and social care are often disregarded or even stigmatized, leading older population to strongly reject diversified forms of older adult care:

*“Many older adults associate nursing homes or senior apartments with unfilial children, believing that entering such institutions means being abandoned.”*

At the same time, respondents believed that under such circumstances, the concept of filial piety constrained the children’s actions, making them hesitant to consider older adult care options beyond family-based care:

*“Many friends say, ‘I would never send my parents to a nursing home!’ What lies behind this sentiment? It stems from the traditional belief that such actions contradict filial piety.”*

#### 3.3.4 Vision for the future of older adult care

Regarding the future of older adult care, people also expressed confidence in the discussions. Responses mentioned the legalization of euthanasia, technology-assisted older adult care, and market-oriented older adult care driven by the silver economy.

##### 3.3.4.1 Openness to euthanasia

Part of the respondents expressed an open attitude toward euthanasia, hoping that China will establish related policies in the near future:

*“If one day I become bedridden for a long period, I hope euthanasia can be legalized. I would rather leave early than suffer from illness and pain, and I don’t want my children to be burdened by me.”*

##### 3.3.4.2 Technology-driven smart older adult care

Several optimistic responses suggested that technological developments—especially the Internet of Things, artificial intelligence, big data, and wearable devices—offer new approaches to addressing older adult care challenges:

*“I feel we are living in a favorable era. I hope that in the next 10–20, or even 20–30 years, artificial intelligence and smart home technologies will become widespread, allowing electronic and intelligent solutions to replace part, or even most, of the human labor. This might offer a way forward.”*

##### 3.3.4.3 Silver economy promoting more comprehensive older adult care services

A large number of respondents expressed confidence in the silver economy:

*“However, I believe that as aging intensifies, the supporting policies, products, and services will surely flourish, offering increasingly diverse options in the future.”*

They believed that, with China’s increasing aging population, a market-oriented economy could become an important force in promoting the improvement of the older adult care service system.

*“Once a market matures, elite leaders will inevitably emerge, enhancing the overall quality and standards of the industry. The older adult care sector is destined to become a major market, following these patterns.”*

## 4 Discussion

This study, based on textual data from public online Q&A communities, analyzed the older adult care practices of China’s only children, represented by urban, young, and highly educated groups. This makes the findings of this study more likely to reflect the perspectives of urban middle-class men. The digital filtering effect has, to some extent, resulted in “missing voices,” as the experiences of rural residents, women, and those with lower educational attainment were underrepresented. These groups may exhibit different caregiving patterns and emotional expressions, and they are likely to face more limited resources, greater economic pressures, and stronger social expectations. Meanwhile, online narratives cannot be independently verified and may include exaggeration, representing a key study limitation.

Overall, the sampled group in this study held a pessimistic view toward caring for their parents, anticipating or already bearing a steadily increasing caregiving burden. This chapter, from the perspective of caregiver burden and social support theory, analyzes the multidimensional dilemmas faced by the group of only children participating in Zhihu discussions in their older adult care practices, as well as the underlying causes. Meanwhile, at the practical level, this chapter focuses on their reflections on social institutions and social culture. These experiential reflections from caregivers provide important insights for the improvement of government policies and the enhancement of social support systems.

### 4.1 Caregiver burden

Our findings are similar to those of scholars such as Shen and Ciobanu (20). When parents require intensive care, the only children in our study sample face a range of risks, including physical fatigue, psychological distress, and disruption to their daily lives (20). This situation mainly occurs during the period when older parents partially or completely lose mobility, and their dependence on their children’s care gradually increases (43). Caregivers experience burdens across multiple dimensions, including financial, physical, psychological, and social participation aspects (44).

Due to the family planning policy, the caregiver burden for China’s only children differs significantly from the “sandwich generation” in other countries, mainly in the following ways. Firstly, a heavier burden: A single adult child is typically responsible for the care of two older parents, which is determined by the “4–2-1” family structure. Secondly, lack of peer support: Siblings are usually the main source of peer support in older adult care, but the one-child policy means these only children have no siblings to share the responsibility (45). Thirdly, occurs on a large intergenerational scale: The number of only children in China is enormous, and the care needs of their parents emerge in a concentrated, large-scale manner (23). This will result in an extreme scarcity of caregiving resources, especially human resources, at the societal level over the coming decades. Therefore, in the sample of this study, only children as caregivers face severe challenges both in sharing care within the family and in accessing care support at the societal level.

Role conflict and role strain as caregivers may be particularly pronounced among China’s only children. According to the scarcity hypothesis, role conflict arises when time and resources are limited and an individual faces conflicting expectation across different roles (46). Individuals who assume caregiving responsibilities inevitably face conflicting demands arising from the different roles they occupy (26). In the participants of this study, we can observe that the role of adult child providing care for aging parents increasingly conflicts with their roles as employees, spouses, and parents raising children. Many are forced to interrupt their studies, work, or personal lives, redirecting time, energy, and financial resources toward parental care. Excelling as a caregiver can come at the cost of being a less attentive spouse, employee, friend, or family member (25). Role conflict generates stress, as resources become scarce or depleted while trying to balance multiple roles. This can negatively affect caregivers’ psychological well-being, physical health, and financial stability (26, 46, 47). Our findings indicate that only children probably experience psychological health issues—such as depression, anxiety, pessimism, and fatigue—while caring for impaired older parents. Although the impact on physical health is less pronounced, it is beginning to emerge. The depletion of financial resources is a particularly prominent concern, especially among only children from rural areas or towns with weaker social security. Additionally, the drain on family labor resources is widespread; even in well-off families of civil servants, shortages of available caregiving labor are a significant issue.

Only children who act as caregivers are a crucial group in an aging society and require urgent attention from scholars and policymakers. They form the backbone of society while simultaneously bearing multiple responsibilities: caring for aging parents, raising children, and fulfilling social and professional obligations. To a considerable extent, their physical and mental health, as well as their level of social engagement, directly affect both the current functioning and the future development of the nation.

### 4.2 Social support

Social support, defined as the emotional and material assistance provided by society to individuals, is a key predictor of caregiver burden (47–49). The role of social support in alleviating caregiver burden is widely recognized, and caregivers who receive greater social support report lower levels of burden (48, 50).

Consistent with Lv’s study on the risks and dilemmas faced by “full-time children,” this study indicates that only children bearing caregiving responsibilities may face risks of insufficient social support and increased social isolation (51). A decrease in social support for caregivers corresponds to a heightened risk of social isolation (48). Empirical studies have shown that caregivers of older adults with chronic severe illnesses, such as Alzheimer’s or Parkinson’s disease, often face insufficient social support during the caregiving process, which increases their risk of social isolation (47, 48). In China, based on traditional Confucian filial values and relatively low levels of social security, the care of older adults with serious illnesses or disabilities is primarily undertaken by family members (52). Among the participants in this study, older parents gradually became disabled, and the caregiving burden fell on their adult children. In some cases, children leave their jobs to provide full-time care, a phenomenon that has become increasingly common in recent years and has sparked widespread discussion as the “full-time children” phenomenon (53). Young and middle-aged adults withdraw from the workforce to devote themselves entirely to caring for older parents, with family and personal expenses largely dependent on the parents’ pensions (51). The phenomenon of full-time children has already led to serious consequences in Japan (54, 55). First, children who provide full-time care for their parents experience varying degrees of social isolation (54). Second, even after the parents’ death, these children face numerous barriers when attempting to reenter the labor market (54). Two generations—the older population in need of care and their adult children unable to work—are socially excluded.

In addition, due to insufficient social support, the respondents of this study were compelled to adjust their life choices, particularly affecting their marriage and fertility decisions (e.g., “3.2.5.3 Remain unmarried and childless”). The findings indicate that some only children choose not to marry to avoid caring for their spouse’s parents, and some choose not to have children to devote their energy to caring for their own parents. We can observe that, in the context of population aging, the lack of social support leads to the intergenerational transmission of family caregiving pressures, which is very likely to exacerbate low fertility to some extent.

### 4.3 Practical improvements

#### 4.3.1 Caregiver-friendly social policies

First, in China, promoting integrated urban–rural and intergroup equitable social security systems is an important component of caregiver-friendly policies. For a long time, access to social security in China has been strictly determined by social status (56). In this study (“3.3.2.3–3.3.2.4” in results section), participants from civil servant or state-owned enterprise families reported that their parents enjoy generous pensions and ample, high-quality medical resources. As a result, even in cases of parental disability, these participants face virtually no caregiving burden (with individual parental pensions ranging from 7,000 to 10,000 RMB per month). In contrast, participants from urban worker or resident families indicated that their parents’ social benefits are sufficient for daily living, but in the event of serious illness or disability, the household cannot sustain itself and must rely on the children for support (with individual parental pensions ranging from 1,000 to 3,000 RMB per month). Participants from rural families face the heaviest caregiving burden, needing to cover parents’ daily expenses (with parental pensions of 100–200 RMB per month); any illness or disability can deal a severe blow to the entire household. These data indicate that the gap between the highest and lowest pension levels can reach 35–100 times. The results of this paper are consistent with the measurement of public data in China (57–60)^2^. Scholars Fang, Yu, and Jiang explicitly pointed out that there are significant differences in welfare and social security levels among various groups, including public-sector employees (such as civil servants, staff of public institutions, and state-owned enterprise workers), private-sector employees, urban residents, and rural residents (61). The unbalanced social security across different groups directly contributes to the heavy caregiving burden borne by only children outside the institutional support system. Reducing these disparities, prioritizing the welfare of vulnerable groups, and moving toward genuine equality in social benefits are essential components of caregiver-friendly policies and an aging-friendly society.

Secondly, the government should introduce policies or relevant legislation, such as caregiver-friendly workplace policies (CFWPs), to implement support programs for caregivers in the workplace. Discussions on Zhihu reveal that many only children report being forced to interrupt their work due to sudden caregiving responsibilities, with work–family conflicts being quite common (e.g., “3.1.3.2 Difficulty balancing personal development and caregiving”). Caregivers who strive to balance these responsibilities are likely to exit the labor market, which for them means financial loss and interrupted personal development; for employers, it results in higher employee turnover and increased organizational costs; and for the country, it leads to a significant portion of the working-age population (26, 62, 63). For these reasons, social policies should focus on how to provide workplace support for family caregivers who are also employed. For working family caregivers, the following measures can be adopted. Firstly, implement paid or unpaid caregiving leave, allowing employees to take time off when needed to care for their relatives (64). Secondly, flexible work arrangements, including remote work, telecommuting, flexible hours, and part-time options, enable caregivers to balance their job responsibilities with caregiving duties (65). Thirdly, monitor and regulate employers to prevent workplace discrimination, protecting employees who may be disadvantaged or face bias due to their caregiving responsibilities (65). For family caregivers who have been providing full-time care for their parents and wish to reenter the workforce, social policies should offer career guidance, job training, as well as relevant internships and employment opportunities (66).

Finally, it is necessary for the government to implement age-sensitive public policies. When older adults enter the middle-old [75–84 years old] and very-old stages [85 and above], and care needs surge, targeted support should be provided to family caregivers (37). In discussions on Zhihu, participants expressed their greatest concern about their older parents becoming ill or disabled (e.g., “3.1.3.1 Insufficient family caregiving manpower”). Empirical research shows that care needs related to aging and functional decline begin to concentrate in the advanced age stages (67). Therefore, social policies should be oriented toward caregivers of the oldest-old, providing targeted support such as caregiving allowances and tax incentives, which can effectively alleviate caregiver burden (68).

#### 4.3.2 Promoting a diverse caregiving culture

Based on the results of qualitative analysis, this study argues that, in the context of population aging and low fertility, China needs to promote a diversified caregiving culture to alleviate the caregiving burden on children. It is noteworthy that the findings of this study differ from those of Gui and Koropecki regarding qualitative interviews on the older adult care intentions of overseas Chinese only children (18). In Gui and Koropecki’s study, all respondents generally endorsed the concept of filial piety, emphasized that they were their parents’ sole caregivers in old age, and regarded caring for their parents alone as their primary responsibility (18). In contrast, the qualitative result of this study indicates that the obligation constraints of filial piety on only children are loosening (e.g., “3.3.3 Critique of Confucian older adult care culture”). The respondents not only reflected on and critiqued traditional filial norms in their discussions but also explicitly expressed their endorsement of a diversified caregiving culture (e.g., “3.2.4 Guidance for older parents,” “3.3.4.1 Openness to euthanasia”). We believe that this difference may stem from two factors. First, most participants in this study had already assumed actual caregiving responsibilities for their parents, directly confronting the practical challenges of older adult care. In contrast, Gui and Koropecki noted that their interviewees were relatively young and had not yet participated in their parents’ care (18). Second, the online forum format provided respondents with a more anonymous and open space for expression, allowing them to reveal genuine thoughts that might be difficult to express in face-to-face interviews.

China has a tradition of filial piety that spans thousands of years, with family-based care long dominating the older adult care system (52). The essence of filial piety lies in children providing support for their parents in old age, personally attending to their needs, and ensuring their well-being (69, 70). In this process, children’s economic and social interests are expected to be sacrificed for the sake of their parents’ later life (71). Under the constraints of this cultural norm, forms of socialized care beyond the family have often been devalued or negatively perceived (72).

However, one consequence of the one-child policy has been the atomization of families and the widespread rise of individualistic culture, both of which have led to questioning of the traditional filial piety culture (73, 74). On one hand, the structure and size of these nuclear families make it difficult to fulfill the filial duties expected in traditional extended family arrangements. On the other hand, since parents could only have one-child, many only children in our study reported having grown up under their parents’ intense care and attention, which fostered a more individualistic feature. In our research sample, both only children and their parents have begun to critique the traditional culture of filial piety. They express a demand for diverse, socialized forms of care and hold more open attitudes toward community-based older adult care, mutual-aid care, palliative care, and end-of-life care. Therefore, at the societal level, there should be a conscious effort to guide the integration of diverse, socialized care systems with traditional filial norms, aiming to achieve a more inclusive and sustainable model of older adult care.

#### 4.3.3 Providing support and services for family caregivers within the community

Results from the qualitative analysis (e.g., “3.1.1 Psychological burden,” “3.1.2 Physical burden”) are consistent with Ornstein and Caruso’s research on family caregiving support systems (75). As untrained family caregivers, only children are likely to experience prolonged psychological stress and health risks, highlighting the urgent need for external support at the social level (75). Communities serve as important sites of social support (76). This study argues that it is necessary to provide comprehensive assistance to only-child caregivers through three key areas: training and counseling, health support, and the development of caregiving support systems (76).

Training and counseling: This includes caregiving skills as well as consultations on relevant laws and policies. Community-based training and counseling can help reduce caregivers’ burdens by equipping them with basic knowledge and skills (77). Communities should also provide social information, referral services, and guidance to help family caregivers access relevant policies, social resources, and legal protections (76).

Health and psychological support: Establish counseling services and support groups for caregivers under long-term stress to alleviate pressure and reduce levels of depression (76). Health check-ups and medical support: Provide regular health screenings and medical subsidies to mitigate health issues caused by caregiving responsibilities (78).

Community-based care support system: Communities should offer home-based care and in-home services to meet the needs of aging in place (79). Establish day-care centers to provide daytime care for older adults, relieving family burdens (80). Develop community care centers to improve long-term and end-of-life care systems (81). Respite care: Provide temporary substitute care to allow caregivers to rest and recover (82). Social worker and volunteer networks: Build comprehensive networks of social workers and volunteers within the community to offer ongoing, systematic support to caregivers (83).

#### 4.3.4 Silver economy–driven care industry

The qualitative results (e.g., “3.3.4.3 Silver economy promoting more comprehensive older adult care services”) indicate that many participants believe that high-quality older adult care services require not only government support but also the effective promotion of market mechanisms (84). Our finding echoes the Chinese government’s policy orientation of “developing the silver economy and enhancing the well-being of older adults (85).”

On one hand, economic forces can facilitate the development of older adult care-focused communities and cities, creating comfortable and comprehensive living environments for older adults in areas such as housing, healthcare, wellness, and leisure (86). On the other hand, the expansion of the silver market provides career transition opportunities for some young and middle-aged adults, allowing them to enter sectors related to wellness, therapy, and older adult care services (87). The development of a care industry driven by the silver economy helps alleviate employment pressures in traditional industries while simultaneously meeting the diverse service needs of an aging society and optimizing the allocation of labor and social resources.

#### 4.3.5 Technological development to reduce caregiver burden

The qualitative results show that the only children participating in the discussion regarded technology as the remedy for labor shortages and uneven service provision, which is also a key reason for their optimism about the future of older adult care (e.g., “3.3.4.2 Technology-driven smart older adult care”). This study further supports the views of Lindema, Kim, Thordardottir, Malmgren, Lethin, and others on the technologization of care, namely that technology-driven approaches should serve as a key means of supporting family caregivers in the new era (88, 89). Through smart care systems, remote health monitoring, and wearable intelligent devices, technological development can not only improve the quality of older adult care services but also reduce the burden on family caregivers (89).

At the level of daily family caregiving, technology can provide intelligent assistance in health monitoring, medication management, and rehabilitation guidance, thereby easing caregiver responsibilities (88). At the structural and community levels, technology can optimize management and integrate resources, offering caregivers personalized and precise support services (88). Therefore, advancing the research and application of smart older adult care technologies is a crucial step in building the older adult care system today and a key means of alleviating caregiver burden.

## 5 Limitations and future research directions

Although this study provides insights into the challenges, actions, and reflections of contemporary Chinese only children in caring for their aging parents, it also has certain limitations.

### 5.1 Limitations of data sources

Although this study collected a large amount of data from the Zhihu platform, it is important to note that the sample may not fully represent the generation of only children in China. On one hand, Zhihu users are predominantly highly educated urban young males, which limits the generalizability of the findings (90). Voices from rural populations, females, and less-educated groups are relatively underrepresented. On the other hand, the use of online narratives carries the risk that their authenticity cannot be independently verified, and exaggeration or fabrication may exist. Therefore, future research could extend to offline settings and encompass a broader range of populations to achieve a more comprehensive and objective understanding.

### 5.2 Limitations of research methods

This study employed grounded theory for qualitative analysis, with a relatively small sample size, making it difficult to generate statistically significant inferences about the broader population. Therefore, when generalizing the findings to a broader population, some bias may exist, and the validity of the conclusions still requires further verification. Future research could rely on national-level surveys to conduct quantitative and life-course studies to address the limitations of this study.

## 6 Conclusion

This study focused on discussions on Zhihu regarding only children caring for their aging parents. Using grounded theory, it constructed a thematic framework encompassing three categories: challenges, actions, and reflections. Our findings suggest that the participants exhibit a neutral-to-slightly pessimistic outlook regarding their future responsibilities; they have begun to confront caregiving pressures and to reflect critically on traditional filial norms. The findings indicate that caregiving pressures are primarily concentrated in families with limited resources, low levels of social security, or parents in poor health. Based on these observations, this study recommends that policymakers focus on high-risk groups to provide targeted and preferential support. The study also suggests that leveraging technology and the silver economy, developing diversified caregiving models, and strengthening caregiver-friendly social support systems and policies could help alleviate the overall caregiving burden on only children.

It should be noted that this study is based on a limited sample of online narratives, and the findings should be regarded as suggestive and exploratory. Given the trends of population aging and low fertility in China, the issue of only children caring for their parents is likely to persist for an extended period. Future research could validate and expand upon the observations of this study on a larger scale.

## Data Availability

The datasets presented in this study can be found in online repositories. The names of the repository/repositories and accession number(s) can be found in the article/supplementary material.

## Footnotes

1 The Communiqué of the Fifth Plenary Session of the 18th Central Committee of the Communist Party of China, adopted on October 29, 2015, stated: “Promote balanced population development, uphold the fundamental national policy of family planning, improve the population development strategy, fully implement the policy allowing each couple to have two children, and actively carry out measures to address population aging.

2 The above figures were estimated by our research team. The following provides the data sources and calculation process. According to the 2024 Statistical Bulletin published by the Ministry of Human Resources and Social Security of China, urban and rural residents (mainly covering farmers) had total fund expenditures of 532.2 billion yuan, with 180.39 million beneficiaries. This allows the calculation of the average annual pension per person: 53.22 billion yuan ÷ 180.39 million ≈ 2,950 yuan/year, or about 246 yuan per month. The bulletin indicates that the total fund expenditure for urban employees (including civil servants) was 6,765.6 billion yuan, with 147.39 million retirees, yielding an average annual pension per person of 6,765.6 billion ÷ 147.39 million ≈ 45,900 yuan/year, or about 3,825 yuan per month. However, these figures do not differentiate between public-sector and private-sector employees. Since social security data for Chinese civil servants is not directly available through public sources, we roughly estimated it using the method of multiplying wages by pension replacement rates. Using employment numbers and salaries from the “Social Administration, Social Security, and Social Organizations” sector in the China Statistical Yearbook 2024 (approximately corresponding to Chinese civil servants and major public institutions), the calculation was: 2,326.68 billion ÷ 19.858 million ≈ 117,000 yuan/year. Applying the 90% pension replacement rate suggested by scholars, the average annual pension for public-sector employees is 117,000 × 90% ≈ 105,300 yuan/year, or 8,775 yuan per month. For private-sector employees, the annual average salary was taken from the National Bureau of Statistics’ 2024 Annual Average Wage of Urban Unit Employees, which is 69,476 yuan. Using a 45% pension replacement rate proposed for private employees, the estimated average annual pension is 69,476 × 45% ≈ 31,264 yuan/year, or about 2,605 yuan per month.
